# Regulation of Sexual Behavior and Health in German Prisons and Forensic Psychiatric Hospitals

**DOI:** 10.1002/bsl.2711

**Published:** 2024-12-16

**Authors:** Hanna H. Hanss, Susanne Bründl, Maria Krasnova, Johannes Fuss

**Affiliations:** ^1^ Institute of Sexual Medicine and Forensic Psychiatry and Psychotherapy University Hospital Schleswig Holstein Kiel Germany; ^2^ Center for Translational Neuro‐ and Behavioral Sciences Institute of Forensic Psychiatry and Sex Research University of Duisburg‐Essen Essen Germany

**Keywords:** forensic psychiatry, prison, sexual health, sexual rights

## Abstract

Individuals housed in prisons or forensic hospitals experience significant restrictions on their sexual rights. There is a lack of data on how sexual behavior and sexual health of institutionalized persons are managed and to what extent they are based on shared guidelines or decisions of the individual staff. Using a standardized online questionnaire, the heads of 35 prisons and 32 forensic psychiatric hospitals across 14 German federal states were surveyed, reflecting the situation of 16,902 inmates and patients. The findings reveal an absence of shared guidelines as well as an institution‐specific approach to sexual behavior and sexual health. Exploratory multiple linear regression identified four variables that predict differences in the regulation of individual's sexual behavior and sexual health: type of institution (prison or forensic hospital), percentage of institutionalized sex offenders, number of housed individuals, and an item concerning the sexuality‐related attitudes of the respondent. Respondents, particularly in the field of forensic psychiatry, expressed a general desire for guidelines. Our study shows that possibilities for sexual expression and sexual health vary strongly, depending on the institution. Therefore, it appears useful and desirable to develop guidelines on how to regulate sexual behavior and sexual health of institutionalized individuals.

## 1 Introduction

Sexual health is not merely the absence of disease, dysfunction or infirmity but requires a positive and respectful approach to sexuality and sexual relationships, as well as the possibility of having pleasurable and safe sexual experiences, free of coercion, discrimination and violence (World Health Organization 2006). It is an integral part of human identity (Sielert 2008) and determines general and mental health (Anderson 2013; Brand et al. 2022). Positive sexual experiences and sexual satisfaction are related to general well‐being (Anderson 2013; Vasconcelos et al. 2022). Thus, sexual rights are grounded in universal human rights (WAS 2014). Article eight of the European Convention of Human Rights (ECHR) provides a right to respect for one's private and family life, also guarding aspects of sexuality (Council of Europe 1950; European Court of Human Rights 1981, 1985). Precedents show that this may also apply to the sexual rights of prisoners (European Court of Human Rights 2007, 2015).

Imprisonment and other forms of detainment, however, severely restrict the sexuality of institutionalized persons (Anex et al. 2023; Bammann and Rademacher 2009; Barth 2015; Borchert 2017; Brand et al. 2022; Döring 2006; Knop and Zimmermann 2023). International studies indicate that prisoners experience poorer mental health compared to the general population (Favril et al. 2020; Fazel and Danesh 2002; Fazel et al. 2016; Gómez‐Figueroa and Camino‐Proaño 2022; Otte et al. 2017; Vicens et al. 2011; Zabala‐Baños et al. 2016), while prisoners who report higher levels of sexual satisfaction tend to have better mental health (Carcedo et al. 2007, 2019). Therefore, maintaining sexual health is also important from a mental health perspective.

Sexuality is often handled as a taboo subject in forensic psychiatry and prison (Dudeck 2019; Hales et al. 2006; Stöver 2016). This is reflected in the laws regulating life in prison and forensic institutions that barely address the topic of sexuality and sexual health. The belief that sexual deprivation is part of the punitive aspect of imprisonment seems to be present in society and among prison staff (Stöver 2016), even though it is in conflict with the rehabilitative aims of imprisonment in countries such as Germany. While sexuality is rarely addressed openly in these institutions, romantic and sexual relationships do exist in forensic psychiatry and prison (e.g., Döring 2006; Landi et al. 2020). Prohibiting sexual contacts does not result in a higher level of safety (Dein et al. 2016), but severely impairs the human rights of institutionalized persons (Poole 2020).

In the German judicial system, institutionalized persons are usually separated by sex or gender. The accommodation of transgender persons is not uniformly regulated (Hillert und Curic 2022). Heterosexual contacts are rare and limited to external visitors. Regular visits in prison take place in supervised settings, in which little contact is possible (Döring 2006). Unsupervised long‐stay visits in apartment‐like rooms are even more rare and often linked to conditions such as compliant behavior or a minimum sentence length (personal communication, November 7, 2024). Few such rooms are available (Borchert 2017). There are no binding legal regulations on long‐stay visits (Klemm 2016). Although there is no legal right to enable sexual encounters, there is an indirect right to a certain amount of privacy as inmates are given a single room and can only share a room, if they agree to do so (Stein, Itzel & Schwall 2005). This does not apply to forensic psychiatric hospitals, where patients are mostly accommodated in shared rooms (Götzl et al. 2023), allowing little privacy. Some forensic hospitals allow sexual contact with external partners in designated visiting rooms (Götzl et al. 2023; Tiwana et al. 2016). Generally, partnerships with external partners are more accepted by staff than partnerships among patients (Bartlett et al. 2010; Tiwana et al. 2016). Sexual contact among patients is, however, often prohibited in these institutions (Anex et al. 2023; Frei and Vallini 2014). In forensic psychiatric hospitals as well as prisons, opportunities for sexual contact are created through the allowance of unsupervised visits between patients or prisoners (Barth 2015; Tiwana et al. 2016). In the prison environment, same‐sex sexual experiences are usually judged and devalued (Bammann and Rademacher 2009). Various studies show, that prisoners that do not conform to the heteronormative prison environment, are more likely to experience bullying, violence, harassment, coercion and sexual assault (Brömdal et al. 2019). This risk is even higher for transgender prisoners (Brömdal et al. 2019). Nacci and Kane (1983) found 12% of prisoners to have had homosexual contact during their previous period of incarceration. Saum et al. (1995) described 2% of prisoners reporting such experiences within the last year. In a more recent sample of *N* = 2018 Australian prisoners, 13% reported to have had at least one homosexual contact in their life. Only 7% reported to have had sex with a male partner inside prison, 2.7% of which stated to only have had homosexual experiences inside prison (Richters et al. 2012). In this sample, 95.1% of prisoners identified as heterosexual, 3.1% as bi‐sexual and 1.3% as homosexual. In comparison, 2%–6% of the general population identify as homosexual or have had same‐sex sexual experiences (Mercer et al. 2013; Smith et al. 2003). Only 0.9% identify as bisexual (Smith et al. 2003). In conclusion, there is a higher prevalence of same‐sex experiences and bisexual orientation among prisoners. Studies also show that there are individuals who report only opposite‐sex experiences prior to institutionalization that are initiated into same‐sex sexual experiences in prison (Richters et al. 2012; Sagarin 1976). Some prisoners use sexual favors to trade for material substitute currencies or the protection of fellow inmates with a more powerful social standing (Borchert 2017; Richters et al. 2012).

The number of prisoners that have been victim of at least one case of sexual violence among inmates or, more rarely, by staff, is estimated between < 0.5% and 20% (Gaes and Goldberg 2004). A German study reported 7.1% of juveniles, 4.5% of men, and 3.6% of women stating to have been victims of sexual violence in prison in the last 4 weeks (Bieneck and Pfeiffer 2012). The majority of forensic psychiatric staff interviewed by Götzl et al. (2023) also reported to have experienced at least one sexual assault between patients or from patients to employees throughout the course of their career. Some hospitals report official procedures in dealing with such situations (Götzl et al. 2023).

The availability of pornographic material and sex toys in forensic institutions is still subject to professional debate (e.g., Johst 2021; Tewksbury and Demichele 2005; Shaddel and Mayes 2023). If permitted, the pornographic magazines accessible in prison sometimes are limited to heterosexual content, discriminating queer prisoners (personal communication, November 7, 2024).

Regarding sexually transmitted diseases, the prevalence of the human immunodeficiency virus (HIV) is significantly higher among persons in prisons than in the average population (Klemm 2016; Kraft and Knorr 2009). Nevertheless, according to German legislation, prisons are not obliged to hand out free contraceptives (Higher regional court [*Oberlandesgericht*] Koblenz 1997) that provide protection. Worldwide, less than one‐fourth of countries are known to have programs to distribute condoms in prison (Moazen and Stöver 2024), even though Butler et al. (2013) found that the availability of condoms does not appear to increase the frequency of consensual or nonconsensual sexual contact, merely the proportion of protected sexual intercourse.

Patients in forensic psychiatric hospitals are often negatively impacted in their sexuality by mental illness and side‐effects of psychopharmacological treatment (Müller and Eckert 2016). Mental health professionals often weigh the patient's right to sexual autonomy against risks of limited ability to consent, unwanted pregnancy, transmission of sexually transmitted diseases, and sexual violence (Poole 2020; Quinn and Happell 2016), for which there is an increased vulnerability among psychiatric patients (Dudeck 2019; Warner et al. 2004). Some forensic psychiatric hospitals address these issues by training their staff on topics of sexuality and partnership, offering sexual education for patients, and providing access to contraceptives and sex‐specific medical check‐ups (Götzl et al. 2023; Tiwana et al. 2016). Nevertheless, there is a lack of specific education and staff training reported by mental health professionals (Carreiro Da Costa Faria E Melo Höfle and Degano Kieser 2014; Dudeck 2019).

How sexual behavior and sexual health are dealt with differs internationally, between federal states, between institutions, and even between wards of the same institution (Anex et al. 2023; Boons, Jeandarme, and Denier 2024; Brand et al. 2022; Steinberg et al. 2012; Taylor and Whiting 2022; Tiwana et al. 2016). Individual studies confirm the existence of written rules for dealing with sexuality in some institutions (Bartlett et al. 2010; Tiwana et al. 2016). These policies often are restrictive, focusing on safety measures or staff behavior (Bartlett et al. 2010; Poole 2020). Additionally, many facilities operate with non‐written implicit norms, that restrict or prohibit sexual activity (Anex et al. 2023; Bartlett et al. 2010; Dein et al. 2016; Krumm et al. 2014; Landi et al. 2020; Ravenhill et al. 2020; Stöver 2016; Tiwana et al. 2016). However, there is a lack of professional consensus on best practices (Anex et al. 2023; Quinn and Happell 2016). Therefore, individual shared practices often develop, describing rules and norms that are verbally communicated between staff or staff and patients (Anex et al. 2023; Bartlett et al. 2010; Landi et al. 2020; Steinberg et al. 2012; Tiwana et al. 2016).

Poole (2020) interviewed British forensic hospital directors (*N* = 10), finding rehabilitative and human rights‐oriented attitudes that would be consistent with enabling patients to pursue sexual expression. Nevertheless, change toward less restrictive policies and practices was perceived to be hampered by a risk aversion that tolerated the prevailing restrictions on patients' human rights under the ECHR (Poole 2020). The forensic hospital employees interviewed by Götzl et al. (2023) unanimously supported the implementation of written guidelines on how to address patients' sexuality and sexual health. Steinberg et al. (2012) and Götzl et al. (2023) have proposed such guidelines for forensic psychiatric institutions. In the German prison system, however, to date there are no comprehensive written policies on managing sexuality, apart from singular aspects regulated by federal legislation.

Previous studies investigating staff perspectives on sexual behavior and sexual health in prisons and forensic psychiatric hospitals are mostly qualitative. A quantitative study examining the German‐speaking justice system was conducted by Kaufmann, Needham, and Simonik (2016). They asked employees from various professions (*N* = 249) about their attitudes toward sexuality in a forensic context. Compared to a British sample, more permissive attitudes were reported (Di Lorito et al. 2020). Actual behavior was not investigated. Boons, Jeandarme, and Denier (2024) surveyed 32 forensic psychiatric wards in Belgium, finding that only 56% of them had hospital level policies concerning the sexuality of patients. No difference was found in relation to security levels.

The qualitative study by Poole (2020) is to our knowledge the only work that specifically interviewed senior staff. It can be assumed that heads of institutions have a multiplicative role in the implementation of implicit and explicit behavioral norms. In the current study, we surveyed heads of prisons and forensic psychiatric hospitals to explore the following questions:

1. How are sexual behavior and sexual health of convicted offenders managed in German prisons and forensic psychiatric hospitals?
2. Assuming there will be variance between institutions, can the permissive or restrictive handling of sexual behavior and sexual health be predicted by institutional or staff‐related variables?
3. Do written policies exist that outline how sexual behavior and sexual health are managed?

## 2 Methods

### 2.1 Procedure and Participants

We conducted a cross‐sectional exploratory expert survey using a standardized and anonymized online questionnaire. Experts were defined through their profession directing a German prison or forensic psychiatric hospital or being named by the direction as an expert representative of said institution.

Following the ethical approval of the ethics committee at Lübeck University, 25 of 30 German federal ministries of law or social affairs supervising prisons and forensic psychiatric hospitals gave their consent to run the survey among the heads of their institutions. From November 2023 to February 2024, we contacted all forensic psychiatric hospitals nationwide and 60.5% of German prisons in 11 (of 16) federal states. Invitations to the online survey were sent via e‐mail, including two scheduled reminders (Van Mol 2017). The survey was conducted using the software QualtricsXM (Qualtrics, Provo (US)). All participants gave informed consent.

### 2.2 Description of the Sample

We initially received 94 responses, equaling a response rate of 53%. 26 incomplete data sets and one solely referring to youth offenders were excluded, resulting in a final sample of 67. Thus, we sampled 28% of the institutions of interest.

Data out of 14 (of 16) German federal states were collected. Participating institutions varied in size (number of institutionalized persons *M* = 252.27, SD = 198.34, range = 1180), sex of institutionalized persons (67.2% male only, 4.5% female only, 28.4% mixed), and percentage of persons convicted of sexual offenses (*M* = 12.28, SD = 14.89, range = 70). The sample consisted of 35 prisons and 32 forensic psychiatric hospitals. Prisons were executing sentences of different lengths and all common forms of incarceration that exist in Germany. Participating forensic psychiatric hospitals worked based on all different possible legal foundations (e.g., § 63 and § 64 of the German penal code) and treated various disorders, most commonly substance use‐related disorders.

The responding staff had a mean age of 51 years (SD = 9.34, range = 37) and a mean of 18 years (SD = 9.56, range = 33.3) experience working in the German justice system. 49% of participants were female, 48% were male. One participant identified as non‐binary and one did not specify.

### 2.3 Instruments

We used a purpose‐designed questionnaire. It consisted of demographics of the respondent (four items), demographics of the institution (eight items), a global assessment on how sexual behavior and sexual health of institutionalized persons is dealt with (two items), a scale on the dealing with of sexual behavior and sexual health of institutionalized persons (21 items), and attitudes of the respondent toward sexuality (five items). Parallel versions were used targeting prisons and forensic psychiatric hospitals.

The questionnaire was designed in an iterative process. Content‐wise, it is based on the existing literature (Di Lorito et al. 2020; Döring 2006; Frei and Vallini 2014; Götzl et al. 2023) as well as a series of preliminary qualitative interviews with employees (*N* = 6) of the northern German justice system who did not participate in the survey. The items on sexual resources exclusively target legal images, pornographic videos, and sex toys. Since sex work has been fully legalized in Germany for workers as well as clients (German prostitution act, 2001), it has been included as well. Subsequently, the questionnaire was reviewed by field‐experienced scientists of different professions (a psychiatrist working in prison, a former prison director, psychologists in forensic research and practice, and others) as well as the cooperating ministries. In response to feedback, the questionnaire was revised multiple times.

For the present analyses, the scale on dealing with sexual behavior and sexual health of institutionalized persons is the core part of the survey. It contained 21 unipolar items exploring possibilities of sexual activity and partnership (seven items), access to resources (six items), and medical care (two items), as well as documentation and professional communication (six items). After indicating the personal level of agreement with a statement on a four‐point Likert scale (“statement applies” [1] to “statement does not apply” [4]), each item was followed by the categorical question as to whether the given response was based on a written policy, shared practice, or professional assumption.

To assess attitudes toward sexuality, three items of the sex positivity scale (Belous and Schulz 2022) were translated into German and then retranslated into English by a bilingual psychologist (“Sexual health is a basic human right”, “Sex is not a taboo subject for discussion”, and “I believe that a healthy sex life is important to everyone”). All other items were designed by the authors. Answers regarding existing written policies were collected through open text responses.

### 2.4 Analyses

All quantitative analyses were performed using the software Jamovi (version 2.3.28.0, The jamovi project, 2022). Firstly, we used descriptive statistics to assess demographics of the sample and individual items. Based on the responses on the main 21‐item scale, a general “permissiveness index” was formed, reflecting the extent to which institutions permit sexual behavior and provide resources to maintain sexual health. We did so by adding item responses and weighting them by the number of questions answered to compensate missing values. Further, we used multiple linear regression to investigate whether the “permissiveness index” can be predicted through other surveyed variables. Predictors were selected through exploratory forward stepwise regression. Akaike information criterion (AIC) and Bayesian information criterion (BIC) were used as stopping criteria, with the BIC being preferred as more conservative criterion (Fahrmeir, Kneib, and Lang 2007; Fox 2016; Harrell 2015).

Further, the frequencies of decisions based on written policy, shared practice, and professional assumption were examined. Due to a lack of normal distribution, Wilcoxon signed rank test and Mann–Whitney *U* test were used for further analyses. Level of significance was set to *α* < 0.05.

## 3 Results

### 3.1 Management of Sexual Behavior and Sexual Health

The descriptive results show variability between institutions in terms of permissiveness toward sexual behavior and sexual health. For all but two items, all Likert options were used (see Table 1). Ceiling effects suggesting uniform agreement among participants, were observed in the items “visits from external partners” (item 1) and “partnerships between inmates or patients” (item 2). Similar distributions were obtained concerning access to sexuality‐related information (item 13), contraceptives (item 14), and sex‐specific medical check‐ups (item 15), as well as equal treatment of patients and prisoners regardless of sexual orientation (item 16). The same applied to the items on dealing with sexual violence between inmates or patients (item 17) and breaches of professional distance between inmates or patients and staff (item 18). In contrast, the items concerning possession of pornographic videos (item 9) and visits by sex workers (item 11) showed floor effects. Mixed responses resulting in bimodal distributions were received regarding the possibility for prisoners or patients to have privacy (item 4), the possibility to be sexually active with external partners (item 6), and the availability of rooms for long‐stay visits by external partners (item 7). Absolute and relative responses for all items are given in Table 2.

**TABLE 1:** Items and descriptives of the scale on dealing with sexuality and sexual health of institutionalized persons, showing sample size, mean, median, standard deviation, minimum and maximum. Responses were given on a four‐point Likert scale from 1 = disagree to 4 = agree.

	Item	*N*	*M*	Median	SD	Min.	Max.
1	Prisoners/patients are allowed to have partnerships with external persons	67	3.84	4	0.510	1	4
2	Prisoners/patients are allowed to have consensual partnerships with other prisoners/patients	67	3.37	4	0.967	1	4
3	Prisoners/patients are allowed to have consensual sex with other prisoners/patients	67	3.04	3	1.107	1	4
4	There are time frames in which prisoners/patients can be sure to be alone and undisturbed	67	2.84	3	0.994	1	4
5	Prisoners/patients are allowed to exchange consensual sex for material goods	67	1.66	1	1.023	1	4
6	Prisoners/patients are allowed to have sex with external visitors	67	2.52	3	1.397	1	4
7	There are rooms for long‐stay visits where prisoners/patients are allowed to have sex	67	2.25	1	1.460	1	4
8	Prisoners/patients are allowed to use sexualized magazines/images	67	3.21	3	0.946	1	4
9	Prisoners/patients are allowed to use pornographic videos	67	1.66	1	1.008	1	4
10	Prisoners/patients are allowed to use sex toys	67	1.94	2	1.043	1	4
11	Prisoners/patients are allowed to use sexual services provided by sex workers	67	1.31	1	0.722	1	4
12	Prisoners/patients have the opportunity to express sexual concerns to staff members	67	3.12	3	0.913	1	4
13	Prisoners/patients have the opportunity to inform themselves about topics of sexuality	67	3.48	4	0.660	1	4
14	Prisoners/patients have the opportunity to receive contraceptives	67	3.54	4	0.876	1	4
15	Prisoners/patients have the opportunity to receive sex‐specific medical check‐ups	66	3.58	4	0.801	1	4
16	The same rules apply to prisoners/patients regardless of their sexual orientation	66	3.72	4	0.692	2	4
17	Sexual assaults among prisoners/patients can be dealt with appropriately	66	3.37	4	0.813	1	4
18	Violations of proximity and distance between staff and prisoners/patients can be dealt with appropriately	66	3.42	4	0.742	2	4
19	The sexuality of prisoners/patients is part of professional communication between staff members	67	2.97	3	0.953	1	4
20	The sexuality of prisoners/patients is part of their individual resocialization/treatment plan	67	2.36	2	1.011	1	4
21	Staff members are trained in sexuality‐related topics	67	2.12	2	0.977	1	4

**TABLE 2:** Absolute and relative responses from the scale on dealing with sexual behavior and sexual health of institutionalized persons. Responses have been dichotomized into agreeing (statement applies or rather applies) and disagreeing (statement rather not applying or not applying).

		*N*	Agreeing		Disagreeing	
1	Partnerships with external partners	67	65	97%	2	3%
2	Partnerships with internal partners	67	54	81%	13	19%
3	Internal sexual partners	67	46	69%	21	31%
4	Privacy	67	42	63%	25	37%
5	Prostitution between inmates	67	15	22%	52	78%
6	External sexual partners	67	35	52%	32	48%
7	Long stay visits of external partners	67	29	43%	38	57%
8	Possession of sexual images/photos	67	53	79%	14	21%
9	Possession of pornographic videos	67	12	18%	55	82%
10	Possession of sex toys	67	18	27%	49	73%
11	Possibility of visits by sex workers	67	6	7%	61	91%
12	Possibility to voice sexual concerns	67	51	76%	16	24%
13	Access to sex‐related information	67	63	94%	4	6%
14	Access to contraceptives	67	58	87%	9	13%
15.	Access to medical check‐ups	66	61	92%	5	8%
16	Discrimination due to sex. Orientation	66	63	96%	3	5%
17	Sexual violence between inmates	66	60	91%	6	9%
18	Distance‐violations toward staff	66	62	94%	4	6%
19	Part of professional communication	67	46	69%	21	31%
20	Sexuality as part of treatment	67	26	39%	41	61%
21	Staff education on sex‐related topics	67	20	30%	47	70%

Using multiple linear regression, relations between the permissiveness index value of the questionnaire on dealing with sexual behavior and sexual health (*M* = 2.83, SD = 0.42, min. = 1.50, max. = 3.81) and a number of person‐specific (gender, age, attitudes toward sexuality) and institution‐specific variables (type of institution, location of institution, percentage of sex offenders, number of housed persons) were explored. Assumptions for multiple linear regression were tested. There was no significant multicollinearity in correlation matrices or variance inflation factor (VIF < 2 for all predictor variables). The scatterplot of standardized residuals and estimated values revealed no obvious violation of the linearity assumption. Visual tests for homoscedasticity, the constant variance of residuals, showed a tendency toward larger errors in high value ranges of the index. QQ‐plot and Shapiro–Wilk test (*p* > 0.05) indicated normal distribution. Independence of errors was checked using Durbin–Watson test (*p* > 0.05).

The following stepwise regression resulted in a model with four predictor variables (see Table 3) that explained a significant amount of variance in the index (*R*2 = 0.677, adj. *R*2 = 0.4579, *F*(4, 61) = 12.32, *p* < 0.001) and produced the lowest values on the information criteria (AIC = 44.1, BIC = 57.0). All selected predictors achieved significance with *p* < 0.001. Other variables were not significant. The highest predictive value was shown by the variable “type of institution”.

**TABLE 3:** Results of the multiple linear regression examining factors that predict variations in the permissiveness index across institutions.

Predictor	Estimate	SE	*t*	*p*	β
Intercept^a^	1.68614	0.18462	9.13	< 0.001	
Institution: Forensic psychiatric hospitals—prisons	0.37922	0.09556	3.97	< 0.001	0.891
Item EZ2 “sex is not a taboo subject for discussion”	0.14680	0.03677	3.99	< 0.001	0.385
Percentage of sexual offenders	0.00982	0.00271	3.63	< 0.001	0.349
Size/number of housed persons	8.47e‐4	2.41e‐4	3.51	< 0.001	0.395

^a^ Represents reference level.

### 3.2 Written Policies

**FIGURE 1:**
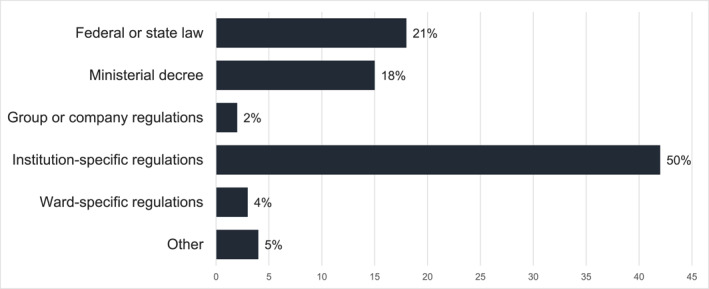
Written policies mentioned by the respondents categorized by administrative levels. Absolute frequencies and percentage of policies in the given category in relation to the total amount of policies mentioned.

A total of 54 institutions (81%) reported having written policies on the sexual behavior and sexual health of inmates or patients. Open text responses revealed institution‐specific policies, such as house rules, policies, work instructions or safety regulations, as the most common (see Figure 1).

On average, 20% of the questions per institution were answered on the basis of written regulations, 67% on the basis of shared practice, and 12% of the questions on the basis of a professional assumption. Further descriptive data are presented in Table 4. For each item of the questionnaire, at least one institution reported a written policy. The possession of pornographic videos (34 institutions, 52%) was most frequently reported as being regulated, followed by the availability of long‐stay visiting rooms (30 institutions, 46%) and the handling of sexual images (29 institutions, 43%). Rarely regulated were the possibility of expressing sexual concerns (1 institution, 2%), professional communication regarding the sexuality of inmates or detainees (3 institutions, 5%), and the possibility of sexual relationships between inmates or patients (4 institutions, 6%). Absolute and relative frequencies for all items are given in Table 5.

**TABLE 4:** Descriptives on the frequency of answers on the main questionnaire based on written policy, shared practice, or professional assumption.

	*N*	Mean	Median	SD	Range	Min.	Max.
Written policies	67	4.12	3	3.70	14	0	14
Shared practice	67	14.04	14	4.36	19	2	21
Professional assumption	67	2.57	1	2.99	10	0	10

**TABLE 5:** Absolute and relative responses from the scale of dealing with sexuality and sexual health of institutionalized persons showing whether decisions were based on written policy, shared practice, or professional assumption.

	Item	*N*	Written policy		Shared practice		*p*. assumption	
1	Partnerships with external partners	67	14	21%	51	76%	2	3%
2	Partnerships with internal partners	67	6	9%	47	70%	14	21%
3	Internal sexual partners	67	4	6%	51	76%	12	18%
4	Privacy	65	10	15%	50	77%	5	8%
5	Prostitution between inmates	66	12	18%	37	56%	17	26%
6	External sexual partners	67	24	36%	41	61%	2	3%
7	Long stay visits of external partners	66	30	46%	34	52%	2	3%
8	Possession of sexual images/photos	67	29	43%	36	54%	2	3%
9	Possession of pornographic videos	66	34	52%	30	46%	2	3%
10	Possession of sex toys	66	12	18%	39	59%	15	23%
11	Possibility of visits by sex workers	67	14	21%	48	72%	5	8%
12	Possibility to voice sexual concerns	66	1	2%	54	82%	11	17%
13	Access to sex‐related information	66	4	6%	53	80%	9	14%
14	Access to contraceptives	66	15	23%	42	64%	9	14%
15	Access to medical check‐ups	65	16	25%	39	60%	10	15%
16	Discrimination due to sex. Orientation	65	14	22%	46	71%	5	8%
17	Sexual violence between inmates	66	8	12%	47	71%	11	17%
18	Distance‐violations toward staff	66	14	21%	45	68%	7	11%
19	Part of professional communication	67	3	5%	52	78%	12	18%
20	Sexuality as part of treatment	66	8	12%	48	73%	10	15%
21	Staff education on sex‐related topics	65	4	6%	51	79%	10	15%

To continue the analysis, a directional Wilcoxon signed‐rank test was used to examine the difference between reported written rules and shared practice per institution (*W* = 61, *p* < 0.001, *r* = −0.941). It was found that the difference between the reported number of written rules and shared practice was significantly less than zero. This supports the hypothesis that the questions about dealing with sexual behavior and sexual health were answered more on the basis of shared practice than on the basis of written regulations.

### 3.3 Desire for Guidelines

On average, respondents tended to report a desire for guidelines on the five‐point Likert scale (*M* = 3.19, SD = 1.21, range = 4). In the subsample of prison heads that tendency cannot be found (*M* = 2.77, SD = 1,29, range = 4), while it prominently shows in forensic psychiatric staff (*M* = 3.66, SD = 0.937, range = 4). The performed Mann–Whitney *U* test (*U* = 328, *p* = 0.003) showed a significant difference in the subsample group means.

## 4 Discussion

### 4.1 Regulations of Sexual Behavior and Sexual Health

In the present study, we aimed to explore how sexual behavior and sexual health of institutionalized persons are dealt with in German prisons and forensic psychiatric hospitals. Therefore, we surveyed heads of these institutions, investigating current practices and written policies. Results show a heterogenous regulation of sexual behavior and sexual health as expected based on existing literature (see Anex et al. 2023; Götzl et al. 2023). On the main questionnaire, no item was answered unanimously with agreement or disagreement. While there seemed to be a consensus in the form of shared practice on some of the surveyed items, other items showed considerable variance. Consequently, the available opportunities and resources related to sexual behavior and sexual health vary, depending onto which facility inmates or patients were assigned.

The majority of participants (97%) agreed or tended to agree that relationships with external or internal partners were permitted. Agreement was less clear in the area of external (52%) or internal (69%) sexual contact, that was prohibited more often. This generally reflects the pre‐described tendency found in the international literature, that external partners are more accepted than internal partners (Bartlett et al. 2010; Tiwana et al. 2016). Tiwana et al. (2016) reported that sexual contact with external partners is possible in the German justice system. Götzl et al. (2023) also reported the existence of visiting rooms in which sexual interaction with external partners is explicitly or implicitly permitted. These observations can be confirmed for some, but not all, of the institutions surveyed. The fact that sexual contact between inmates or patients was reported as more frequently permitted than sexual contact with external partners contradicts previous literature, where they are described as prohibited, tacitly tolerated (Tiwana et al. 2016) or unregulated (Götzl et al. 2023). The discrepancy could be caused by the limited samples of previous qualitative studies. It is conceivable that internal sexual contacts are more easily possible, while contacts with external partners are preceded by considerable institutional effort. Future studies should explore further how these sexual contacts between inmates or patients are regulated and whether this allowance is only relevant for same‐sex sexual contacts, given the strict segregation by gender or sex.

Concerning resources, 79% of institutions reported that they allow or tend to allow the use of sexual images. Pornographic videos were permitted by only 18%, sex toys by 27%, and the use of sexual services by sex workers by only 9% of institutions. This shows inconsistent regulations toward inmates' and patients' sexual behavior.

Most institutions (76%) considered the opportunity to express sexual concerns and receive information on sexuality issues to be a given (94%). The provision of sexual education has already been reported by Tiwana et al. (2016). The nature, availability and accessibility of these services may still vary widely and should be investigated more specifically in future research.

The majority of participants agreed or tended to agree with the provision of contraceptives (87%) and sex‐specific medical check‐ups (92%) of any kind. When asked, 84% of the institutions that agreed stated that they provide contraceptives free of charge. However, in 16% of institutions this was not the case, which is striking given the undeniable medical benefits of such preventive measures. North Rhine–Westphalia obliged prisons to offer condoms and water‐based lubricant to prevent the transmission of sexually transmitted diseases in 1998 (Ministry of the interior and justice North Rhine‐Westphalia 1998). However, this is not standard in other German federal states and thus gives room for individual solutions. Preventive gynecological or urological check‐ups were offered by the institutions interviewed by Götzl et al. (2023). Still, the accessibility of these varied considerably between hospitals. This highlights, that sexual health is not necessarily seen as a human right in some institutions of the German justice system, not even in the crucial aspect of prevention of sexually transmitted diseases or basic medical care.

Most respondents agreed that their institutions can appropriately cope with violations of distance toward staff (94%) and sexual violence among inmates or patients (91%). Those items however do not reveal to what extent inmates, patients or other staff perceive those situations as well‐handled. Nevertheless, the information is congruent with the findings of Götzl et al. (2023), who reported official procedures for dealing with sexual violence that could also be applied by the employees interviewed. Staff training on dealing with sexuality was confirmed by only 30% of participants. This supports the former finding that these are offered in some, but by no means all, institutions (Tiwana et al. 2016).

In summary, the findings of existing qualitative studies and the quantitative data collected as part of this study clearly show a heterogeneous and presumably institution‐specific approach to sexuality of institutionalized persons. Explorative multiple linear regression explained around 46% of variance in dealing with sexual behavior and sexual health between institutions. Significant predictors were type of institution (prison or forensic psychiatric hospital), number of persons institutionalized, percentage of sex offenders and agreement on the attitudes to sexuality item “sex is not a taboo subject for discussion”.

The type of institution explained most of the variance, with forensic psychiatric hospitals on average following a more permissive approach to sexual behavior and sexual health of their patients. This could be caused by the differing legal foundations and societal mandates. In contrast to the prison system, German forensic hospitals are often privatized, which results in different administrative dynamics (du Mesnil de Rochemont 2024). At a conceptual level, forensic hospitals are not only assigned by state laws to reintegrate, but moreover to offer medical treatment. In contrast to prisons, these clinics are usually led by psychiatrists and resemble general psychiatric hospitals in structure. While medical care and prison management are separate tasks in prisons, in forensic hospitals they both are simultaneously carried out by the heads of institutions (Konrad 2024). Further research could analyze the differences in managing sexual behavior of institutionalized persons between German prisons and forensic psychiatric hospitals.

Contrary to the socio‐political intuition that a high proportion of sex offenders could lead to more restrictive approaches in dealing with sexual behavior and sexual health, a positive correlation was found between the permissiveness index value of the questionnaire and the percentage of sex offenders in a facility. Frequent work with sex offenders could create a more open atmosphere through the need to deal with sexuality and to examine the therapeutic value of established prohibitions.

The correlation between the number of inmates and the permissiveness index has to be interpreted with caution. The number of institutionalized persons is not normally distributed. It also showed outliers, particularly at the upper end of the scale, that have a statistical leverage effect. Nevertheless, larger institutions could be more likely to have long‐stay visitation rooms that allow external sexual partners to visit. This could have systematically generated higher scores on the questionnaire used.

The attitude item “Sex is not a taboo subject for discussion” was the only variable in relation to the respondent that produced a statistically significant correlation with the index value of dealing with sexual behavior and sexual health. Respondents who worked in facilities with a higher index score were more likely to agree with this statement. Due to ministerial restrictions, we were not able to use an entire validated scale on attitudes toward sexuality. The used single item therefore must be viewed solely exploratory as a potential indicator for personal sexual morals.

We found that current practice in dealing with sexual behavior and sexual health of institutionalized persons is based significantly more on shared practice, than on written policies. For all aspects addressed in the main questionnaire, at least one, but no more than half of the institutions reported written policies. The analysis of the stated written regulations revealed guidelines at various structural levels (see Figure 1). By far most of them were institution‐specific rules, regulations, and concepts. This can explain the inhomogeneous picture found in dealing with sexual behavior and sexual health, as well as the rather low number of written regulations reported. Previous qualitative studies already point to a lack of written rules and suspected inconsistencies in existing regulations (Anex et al. 2023; Bartlett et al. 2010; Götzl et al. 2023; Tiwana et al. 2016).

Additionally, we also found a subjective need for guidelines primarily in the subgroup of forensic hospitals. Systematic professional differences between the subsamples, for example the convention of guideline‐based work in medicine, could explain those results.

Overall, our German sample revealed institutional inequalities, for example in the accessibility of sexuality‐related medical care or sexual resources. Based on the result, that in at least half of the surveyed institutions, decisions on matters of sexual behavior and sexual health are not made on basis of written policies, the question of necessity for such regulations arises. Inconsistent practices are problematic because sexual health is a human right. Violations of this right should be based on common rules or existing guidelines that are, at best, evidence‐based. The German justice system follows a rehabilitative aim, to provide conditions, that resemble the extramural world as strongly as possible and prepare institutionalized persons for the outside‐life. This as well demands, that restrictions, including those targeting sexual behavior, should be well‐reasoned. On the other hand, a permissive stance toward the sexuality of institutionalized persons might pose questions about topics such as unwanted pregnancy, sexually transmitted diseases, inadequate sexual behaviors, and sexual violence, that need to be discussed. However, sexual expression should not depend on individual institutional structures or attitudes and sexual morals of the responsible staff, which seems to currently be the case. There is an urgent need for professional discourse on the risks and benefits of institutionalized persons' sexual rights and sexual health in order to build professional consensus.

### 4.2 Limitations and Further Research

The present study was subject to bureaucratic hurdles. Due to the double role in authorizing research and supervising the prison system that is being researched, the German ministries of justice have a monopoly on forensic research targeting their institutions. The resulting lack of possibilities for independent forensic sex research has already been highlighted by previous authors (Barth 2015; Knop and Zimmermann 2023). Due to federal responsibilities, 31 research requests were submitted with varying procedures. The design of this study was adapted to meet requirements of the responsible institutions. Conducting the survey in cooperation with the supervising ministries might have generated specific response tendencies, for which we cannot fully control.

Due to the research topic, social‐desirability bias and none‐response bias are likely. The heads of institutions that did not take part in the survey, could differ systematically from those who did. To minimize those effects, we encouraged all eligible participants to take part in the survey, pointing out anonymity and confidentiality.

Due to the absence of pre‐existing standardized scales, we developed a purpose‐designed questionnaire. All items were considerately phrased and underwent extensive revisions. Still, the wording of individual items is debatable. Some require a subjective assessment (e.g., if there is an adequate way to cope with incidents of sexual violence), while other are rather objective (e.g., if pornographic videos are allowed). Respondents' intuitive interpretations of the expressions used (e.g., sexual images) could have varied, because no detailed definitions were given. For further scientific use it seems sensible to create a revised version of the questionnaire.

The results of our multiple linear regression should be considered explorative. More extensive data are needed to confirm correlations, especially in the areas of staff attitudes toward sexuality.

Concerning the research design, a single participant's response does not have to accurately reflect the current state of the institution. A lack of knowledge of regulations or a limited view on current practices could have led to distorted answers. This study is also limited by the focus on the perspectives of senior staff. We chose the population of prison and forensic hospital heads to firstly survey on the top level of decision‐making. Little is known on the perspectives of other employees working in closer contact with inmates or patients, as well as the needs and opinions of inmates and patients themselves. Further studies could also focus the views on perspectives of incarcerated woman, sexual offenders, transgender prisoners, or different correctional settings.

The researched written policies were provided through self‐report. Interesting results could also be obtained by gathering the relevant documents for qualitative analyses.

## 5 Conclusion

This study highlights significant inconsistencies in the management of sexual behavior and sexual health across German prisons and forensic psychiatric hospitals. The findings reveal a lack of standardized guidelines, leading to varied practices that result in unequal opportunities for inmates and patients. This could especially disadvantage those who are of sexual or societal minorities. Sexual health, a human right, is not uniformly protected, with notable differences in the acceptance of sexual relationships and the availability of resources such as contraceptives, sexual images, and educational services. Forensic psychiatric hospitals generally adopt more permissive approaches compared to prisons, likely due to their distinct legal foundations and administrative structures. The lack of formal written policies in many institutions, coupled with a reliance on shared practices, exacerbates the inconsistency in handling sexual health issues. This variability underscores the need for comprehensive, evidence‐based guidelines to ensure that sexual health management is consistent and equitable across all institutions.

## Conflicts of Interest

The authors declare no conflicts of interest.
